# Homoharringtonine in the treatment of acute myeloid leukemia: A review

**DOI:** 10.1097/MD.0000000000040380

**Published:** 2024-11-01

**Authors:** Siyu Shen, Haifeng Zhuang

**Affiliations:** a The First School of Clinical Medicine, Zhejiang Chinese Medical University, Hangzhou, Zhejiang, P.R. China; b Department of Clinical Hematology and Transfusion, The First Affiliated Hospital of Zhejiang Chinese Medical University (Zhejiang Provincial Hospital of Chinese Medicine), Hangzhou, Zhejiang, P.R. China.

**Keywords:** acute myeloid leukemia (AML), homoharringtonine (HHT), mechanism

## Abstract

Acute myeloid leukemia (AML) is a hematological malignancy characterized by the accumulation of immature myeloid precursor cells. Over half of AML patients fail to achieve long-term disease-free survival under existing therapy, and the overall prognosis is poor, necessitating the urgent development of novel therapeutic approaches. The plant alkaloid homoharringtonine (HHT), which has anticancer properties, was first identified more than 40 years ago. It works in a novel method of action that prevents the early elongation phase of protein synthesis. HHT has been widely utilized in the treatment of AML, with strong therapeutic effects, few toxic side effects, and the ability to enhance AML patients’ prognoses. In AML, HHT can induce cell apoptosis through multiple pathways, exerting synergistic antitumor effects, according to clinical and pharmacological research. About its modes of action, some findings have been made recently. This paper reviews the development of research on the mechanisms of HHT in treating AML to offer insights for further research and clinical therapy.

## 1. Introduction

Acute myeloid leukemia (AML), the most prevalent type of acute leukemia in adults, is a highly heterogeneous hematologic malignancy characterized by massive abnormal proliferation of primitive cells and bone marrow infiltration.^[1,2]^ Despite improvements in our knowledge of the molecular heterogeneity and etiology of AML over the past 40 years, the standard therapy has not improved all that much. The standard of care, consisting of hematopoietic stem cell transplantation and cytarabine-based chemotherapy, has an estimated 5-year survival rate of 62% for patients diagnosed before the age of 50, 37% for patients between the ages of 50 and 64, and only 9·4% for patients 65 and above.^[3]^ And patients with AML currently have a 5-year relative survival of 30.5%.^[4]^

Homoharringtonine (HHT), an alkaloid extracted from cephalotaxus, has a molecular weight of 545.65 and a molecular formula of C_29_H_39_NO_9_.^[5]^ HHT prevents further protein synthesis by fixing to ribosomes and blocking the extension of the nascent peptide chain. It has also been shown that HHT may induce leukemia cell apoptosis by inhibiting the production of proteins related to apoptosis.^[6–8]^ After 2 or more tyrosine kinase inhibitors failed to treat chronic or accelerated CML, the U.S. Food and Drug Administration approved HHT in October 2012.^[9,10]^ In China, HHT has been utilized for the treatment of acute myeloid leukemia (AML) for nearly 4 decades.^[11]^ Clinical studies have demonstrated that HHT, when combined with other agents in pretreatment regimens such as the homoharringtonine-cytarabine-granulocyte colony-stimulating factor scheme, which includes HHT with low-dose cytarabine and granulocyte colony-stimulating factor, and the HA scheme combining HHT with cytarabine, has achieved promising results in treating refractory and relapsed AML, leading to hematological remission in the majority of patients.^[12]^ In 2006, a research team pioneered an HHT-based clinical study in Zhejiang province, employing an homoharringtonine-cytarabine-aclarubicin (HAA) induction chemotherapy regimen for newly diagnosed AML, which resulted in an 83% complete remission rate.^[13]^ Subsequently, a multicenter phase 3 trial confirmed that the HAA regimen could serve as an alternative induction therapy for untreated AML, particularly suitable for patients with favorable and intermediate cytogenetic profiles.^[14]^ The HAA induction chemotherapy regimen, anchored by HHT, was incorporated into the expert consensus for first-line treatment of AML in China in 2009.

It was first discovered that HHT can attach to a specific location on the ribosome to limit protein synthesis, especially affecting protein content with a short half-life, including c-myc, myeloid cell leukemia sequence 1 (Mcl-1), cyclin D1, and so on.^[15–17]^ It is believed that the effect on these proteins can cause changes in the downstream apoptosis-related proteins, such as BIM, Bid, Bik, Puma, and so on.^[18]^ Functional inhibition of HHT, related to the BCR::ABL1 protein, has also been reported,^[19]^ and it is also related to the inhibition of Akt phosphorylation and Jak-stat signaling pathway.^[20–23]^ In the present review article, our discussion primarily encompasses the therapeutic mechanisms of HHT for AML, as well as its synergistic effects when used in combination regimens.

## 2. Mechanisms of HHT action in AML

Abnormal changes in cell metabolism and molecular genetics are closely linked to the prognosis of AML. The pathogenesis of AML is a complex process involving multiple factors and pathways, among which epigenetic regulation is widely involved in cell cycle regulation, DNA replication, damage and repair, cell proliferation, differentiation and apoptosis, and other important life activities, which play a key role in the occurrence, progression, and prognosis of AML.^[24]^ Novel studies have demonstrated that HHT induces AML cell apoptosis by involving multiple factors and mechanisms, mainly by targeting various signaling pathways, inducing related protein degradation, affecting cell metabolism, regulating leukemic stem cells, etc.

### 2.1. NF-KB signaling pathway

**Figure 1.**
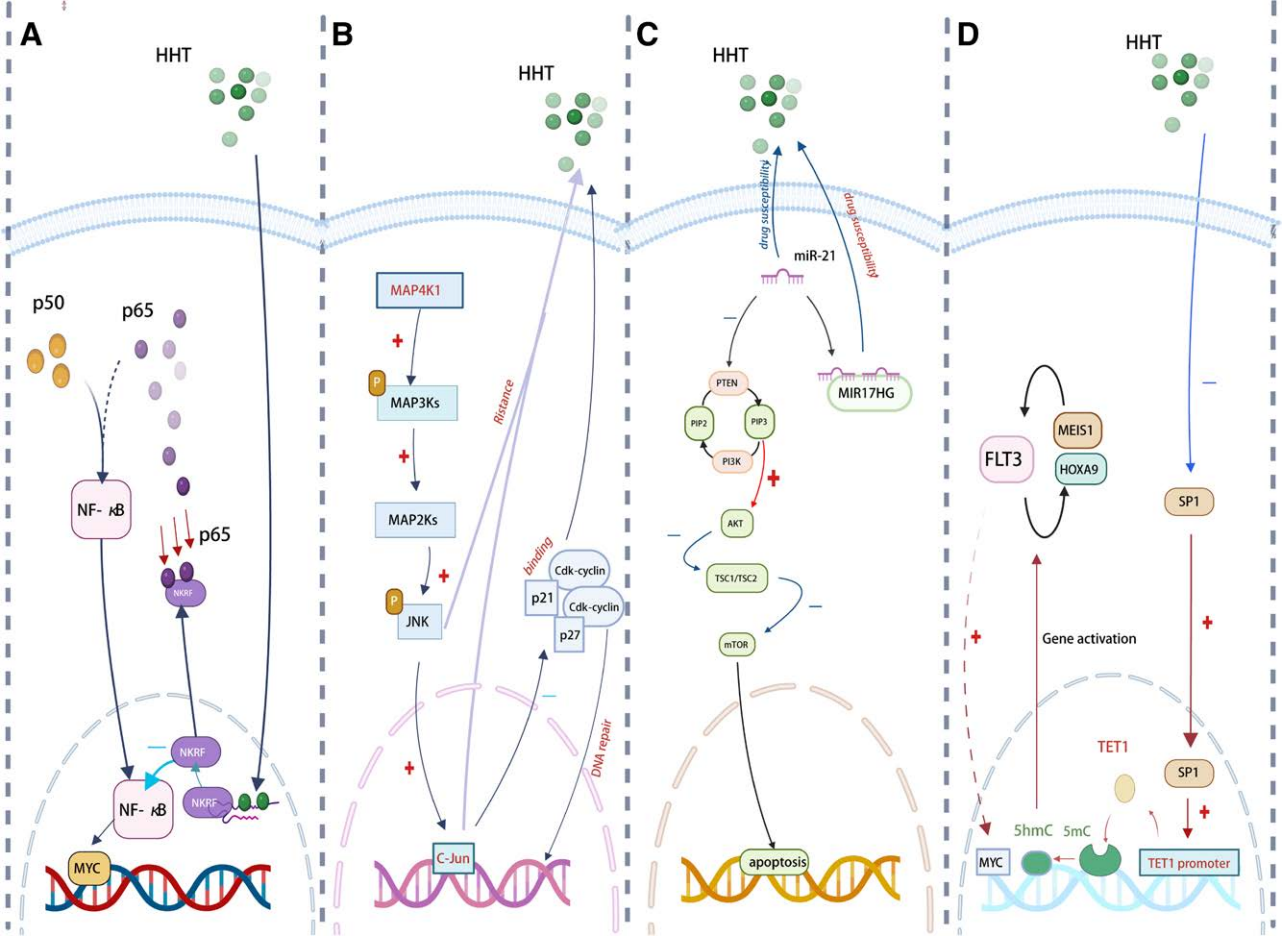
(A) Homoharringtonine (HHT) directly binds the nuclear factor-kappa B (NF-KB) repression factor (NKRF) through the second double-strand RNA-binding motif (DSRM2), a nuclear localization signal for NKRF. This attenuates the transactivation activity of p65 on the MYC gene by strengthening the p65–NKRF connection and interfering with the development of the p65–p50 complex. (B) Mitogen-activated protein kinase kinase kinase kinase 1 (MAP4K1) is mainly mediated through the JNK-JUN and DNA damage/repair pathways, which regulate acute myeloid leukemia (AML) cell cycle progression through P21/P27 proteins and ultimately affect AML progression and HHT resistance. (C) MIR17HG mRNA expression level was decreased in AML and could regulate the miR-21/PTEN axis to promote HHT-induced apoptosis of AML cells and increase drug susceptibility of HHT. (D) HHT can affect AML cell proliferation (MYC and targets) and differentiation (HOXA9 and MEIS1) via binding to the transcription factor specificity protein 1 (SP1). The dioxygenase ten-eleven translocation 1 (TET1) and the receptor tyrosine kinase FLT3 are the main participants in this pathway.

Nuclear factor-kappa B (NF-KB) plays a significant role in leukemogenesis and is a critical regulator of cancer development and inflammation.^[25]^ According to a study, HHT exhibits dual effects in t(8;21) leukemia, suppressing leukemia-initiating cells and downregulating MYC pathway-associated gene expression.^[26]^ The second double-strand RNA-binding motif (DSRM2) was used to identify the nuclear localization signal for the NF-KB repression factor (NKRF). Following several deletion and mutagenesis studies, binding sites in the DSRM2 domain directly targeted by HHT are K479 and C480 amino acids. HHT enhances the p65–NKRF connection, HHT promotes the interaction between p65 and NKRF, inhibits the formation of the p65–p50 complex, and sequesters NKRF from the nucleus, including nucleoli, to the cytoplasm by occupying the DSRM2 domain^[27,28]^ (Fig. 1A). As a result, the ability of NF-kB to transactivate the MYC oncogene is reduced. One gene that is frequently altered and/or overexpressed in t(8;21) AML is KIT, which is strongly downregulated by HHT in addition to MYC.

This mechanism of HHT inhibition of AML cells may be special. Patients who have MYC and KIT overexpression may therefore respond well to HHT therapy. HHT may enhance the efficacy of the current chemotherapy regimen for AML, particularly in subgroups with an activated NF-KB-MYC pathway, by inhibiting both protein synthesis and the NKRF-MYC regulatory axis.

### 2.2. MAPK and DNA damage/repair pathways

The serine/threonine kinase family of proteins includes mitogen-activated protein kinases (MAPKs), which regulate embryogenesis, cell differentiation, proliferation, and cell death pathways.^[29]^ Hematological cancers are also intimately associated with the MAP kinase signaling pathway.^[30]^ The lymphatic and myeloid lineages have significant levels of expression for the upstream MAPK, mitogen-activated protein kinase kinase kinase kinase 1 (MAP4K1). Previous findings suggested that MAP4K1 knockdown could slow the growth of AML by upregulating the activation of P21 and P27 while upregulating the activities of JNK and JUN.^[31–33]^ Lanz MC et al employed GSEA and KEGG analysis to demonstrate that DNA damage and repair pathways related to cell cycle activities were drastically altered following MAP4K1 overexpression and knockdown, further elucidating the precise regulatory pathways connected with MAP4K1 in AML.^[33]^ Modifications in downstream pathways were brought about by variations in MAP4K1 expression levels, and these changes were congruent with the final phenotype. Ling et al explored mechanisms of HHT resistance by comparing gene expression in HHT-resistant and wild-type AML cell lines. Their findings highlighted a distinct expression pattern for MAP4K1, correlating its levels with HHT sensitivity across multiple AML cell lines. In addition to regulating HHT resistance, MAP4K1 functions as a stand-alone prognostic indicator for AML.^[34]^ Collectively, the research results demonstrated that MAP4K1 modulates AML progression and drug resistance through MAPK and DNA damage/repair pathways (Fig. 1B).

### 2.3. miRNA-21/PTEN signaling pathway

The expression of microRNAs (miRNAs) can be regulated by long noncoding RNAs, which are transcripts longer than 200 nucleotides.^[35]^ Small RNA molecules called miRNAs, which have about 22 nucleotides, are crucial for immunity and tumor development. Studies show that miRNAs are involved in the pathogenesis and drug resistance mechanisms of AML.^[36]^

An oncogene in AML has been identified as long noncoding RNA MIR17HG.^[37]^ A further study found that the well-known tumor-suppressive miRNA miR-21 could target PTEN and cause HHT resistance.^[38,39]^MIR17HG may control the miR-21/PTEN axis to modify AML cell chemoresistance. According to the findings of the present study, MIR17HG was downregulated in AML, and its overexpression may have an additional beneficial effect on the HHT-induced death of AML cells by sponging miR-21, which might then upregulate PTEN^[40]^ (Fig. 1C). The aforementioned findings indicated that MIR17HG might increase the chemosensitivity of AML cells in HTT-treated AML, hence suppressing tumor growth.

### 2.4. SP1/TET1/5hmC signaling pathway

There is growing evidence that modifications in the epigenetic machinery can affect the chromatin structure and gene expression, which can then give rise to malignancies. Changes in DNA methylation and histone alterations are hallmarks of epigenetic dysregulation during tumor development.^[41]^

The family of methylcytosine dioxygenases known as the ten-eleven translocation (TET) proteins, which includes TET1/2/3, converts 5 methylcytosine to 5-hydroxymethylcytosine (5hmC), resulting in active or passive DNA demethylation.^[42]^ Due to its major oncogenic role in the pathogenesis of different AML subtypes, TET1 is a potential therapeutic target for the treatment of the disease.^[43–46]^ According to a study by Li et al, HHT has powerful anti-AML effects both in vitro and in vivo. By directly interacting with specificity protein 1 (SP1), blocking SP1’s function in the transcriptional regulation of TET1 expression, and lowering overall levels of 5hmC, it also modifies the DNA epigenome.^[47]^ Furthermore, they showed that HHT-SP1/TET1/5hmC axis exerts specific regulatory effects on FLT3, which means that HHT therapy significantly inhibits the FLT3/MYC pathways^[48]^ (Fig. 1D). Samples of human primary FLT3-ITD AML cells regularly show a very high sensitivity to HHT therapy. All of the data point to the considerable therapeutic potential of HHT-based regimens for the treatment of AML, especially in cases where FLT3 mutations are present. They also point to a hitherto unidentified mechanism that involves HHT-induced 5hmC decrease in the treatment of AML.

### 2.5. Inhibition of P-eIF4E

The oncoprotein eukaryotic translation initiation factor 4E (eIF4E), which binds to the cap of messenger RNA (mRNA), is crucial for the initiation and progression of cancer.^[49]^ However, phosphorylation of eIF4E is necessary for tumor formation, and AML cells in particular need phospho-eIF4E (p-eIF4E).^[50,51]^ In particular, a study revealed that p-eIF4E could be effectively inhibited by using small molecule inhibitors of MNK, which phosphorylates eIF4E.^[52]^

**Figure 2.**
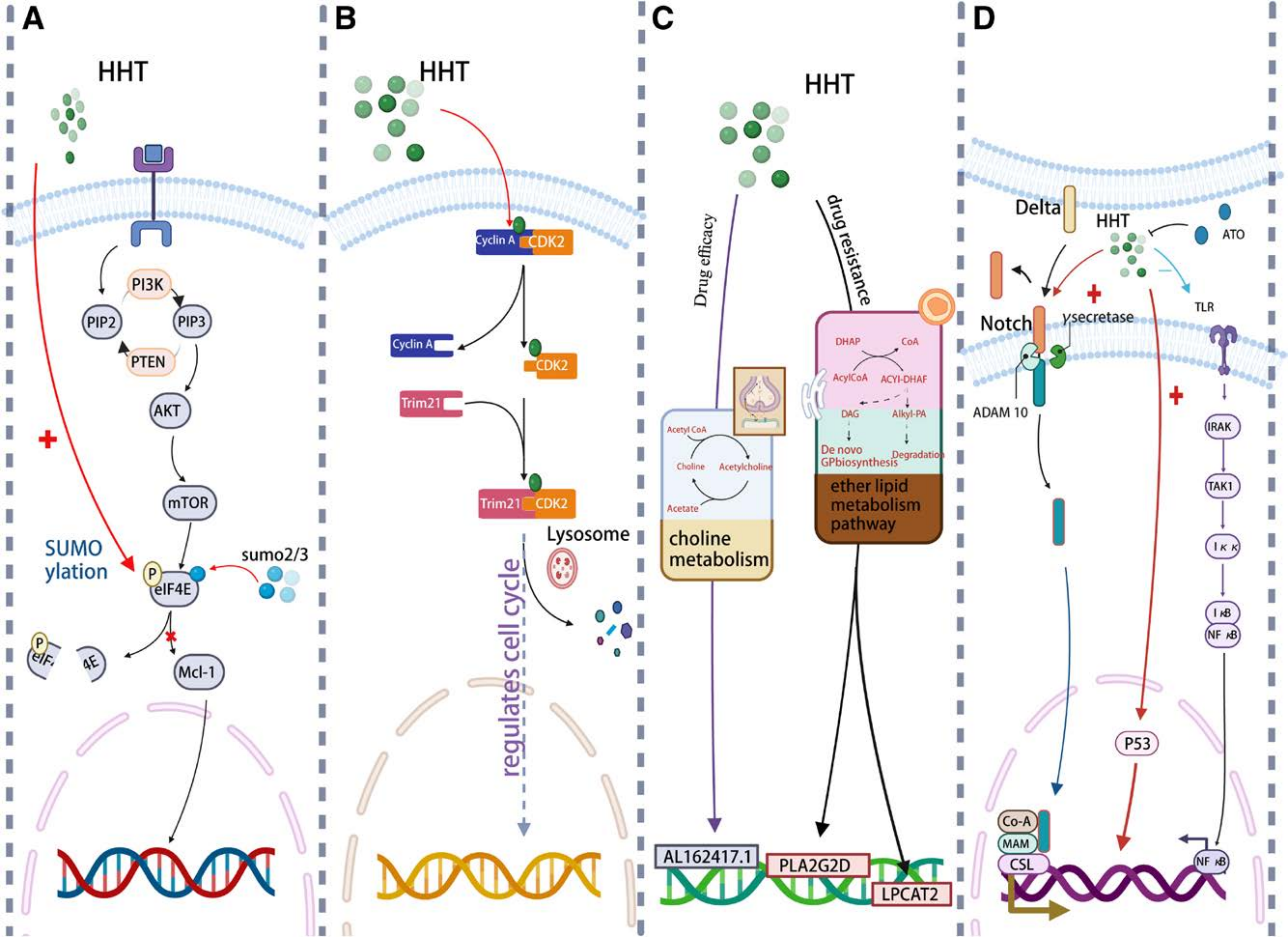
(A) Homoharringtonine (HHT) causes phosphorylated eukaryotic initiation factor 4E (p-eIF4E) to SUMOize more fully. Furthermore, the activity of Mcl-1, the target molecule of HHT-mediated p-eIF4E degradation, can be significantly decreased. (B) The relationship between cyclin-dependent kinase 2 (CDK2) and its partners was broken when HHT attached to the CDK2 protein. Subsequent interaction with tripartite motif 21 (Trim21) led to the autophagy-lysosome system-mediated degradation of the CDK2 protein. (C) The choline metabolism route is intimately linked to the HHT drug efficacy mechanism, and AL162417.1 has been identified as a critical gene. The ether lipid metabolism route is intimately linked to the HHT resistance mechanism, and PLA2G2D and LPCAT2 have been identified as key genes. (D) Notch, P53, and NF-KB signaling pathway-related molecules might be markedly upregulated by HHT alone. In contrast to the considerable suppression of HHT-induced activation of the NF-KB pathway, arsenic trioxide (ATO) coupled with HHT further increased P53.

HHT can potently reduce the proliferation of a specific group of primary leukemia cells and AML cells that exhibit high levels of p-eIF4E by inducing apoptosis both in vitro and in vivo. HHT leads to enhanced SUMOylation of p-eIF4E and HHT-mediated degradation of p-eIF4E can greatly reduce the activity of its target molecule Mcl-1^[53,54]^ (Fig. 2A). The results imply that HHT may be the first medication of its kind that targets p-eIF4E. One can be tempted to hypothesize that this particular inhibitory impact of HHT on p-eIF4E might be further utilized for the creation of fresh anticancer treatment approaches.

### 2.6. An autophagic degradation mechanism of CDK2 protein

The crucial function that cyclin-dependent kinase 2 (CDK2) performs during cell cycle progression is its best-known characteristic. This member of the CDK family is involved in G1/S phase transition, modulation1 of G2 progression, and DNA synthesis.^[55]^ CDK2 becomes active when it assembles into a heterodimeric complex with any of its 2 regulatory partners, cyclins A or E. CDK2’s monomeric form, like that of many other CDKs, is inactive.^[56]^ It appears that CDK2 activity and the activity of its regulatory subunits are crucial elements in the development of oncogenesis.^[57]^

Two potential druggable pockets have been identified by Zhang et al at the protein–protein interaction interface (PPI) between CDK2 and cyclin A.^[58]^ To rule out HHT’s high affinity for PPI and its strong disruption of the connection between CDK2 and cyclin, they conduct a LIVS in silico. Additionally, they show that HHT can interfere with interactions between CDK2 and its cyclin partners in addition to directly binding to CDK2’s PPI site. Moreover, this interaction both prevented CDK2 from functioning and induced cancer cells to degrade it. Additionally, they demonstrate how the tripartite motif 21 in HHT causes autophagic degradation of the CDK2 protein in cancer cells. Thus, these findings point to a CDK2 protein autophagic degradation process and offer a potential treatment option for malignancies that are CDK2 dependent (Fig. 2B).

### 2.7. Changes in metabolic signatures of AML cells

Cancer is characterized by the dysregulation of cellular metabolism.^[59]^ In AML, metabolic abnormalities have a significant role in both disease development and therapy resistance.^[60,61]^ Choline metabolism has been linked to the mechanism of HHT effectiveness in AML, with AL162417.1 serving as the primary candidate gene. The key resistance candidate genes are PLA2G2D and LPCAT2, and the resistance mechanism is connected to ether lipid metabolism^[62]^ (Fig. 2C). In addition, several recent investigations have demonstrated that inhibiting glutamine metabolism or absorption can have antileukemic effects in AML.^[63,64]^ In a variety of distinct AML cell lines, a glutaminase inhibitor reduces glutathione levels while increasing mitoROS and apoptosis. HHT could improve antileukemic activity in AML both in vitro and in vivo when paired with the glutaminase inhibitor CB-839.^[65]^

### 2.8. The killing of leukemia stem cells

Leukemia stem cells (LSCs) are a distinct subgroup of AML cells. LSCs of AML are a subset of CD34-positive and CD38-negative cells, according to the current paradigm, and they may be recognized by a number of molecular markers.^[66]^ Currently, it is widely accepted that LSCs are the cause of AML, making the elimination of LSCs crucial.

Arsenic trioxide (ATO) and HHT have been shown to kill U937 cells synergistically in previous research, and HHT has also shown the capacity to kill LSCs.^[67,68]^ According to studies, combining HHT and ATO had a higher effect in inducing cell apoptosis and halting cell cycle, while also considerably lowering the number of LSCs, compared to either HHT or ATO alone.^[69]^ The Notch, P53, and NF-B signaling pathways’ associated components may be considerably upregulated by HHT alone, according to high-throughput mRNA sequencing. When combined with ATO, HHT increased P53 even more, while dramatically suppressing HHT-induced NF-B pathway activation (Fig. 2D). The changed protein expression in the aforementioned pathways was confirmed by western blot analysis, which also showed that gamma secretase inhibitor could reverse these effects. When HHT and ATO were combined, the LSC burden and LSC marker expression were both significantly reduced in vivo. This provides proof that HHT and arsenic together can kill LSCs both in vitro and in vivo while also identifying the underlying process and indicating a potentially effective treatment approach.

## 3. Mechanisms of HHT synergistic therapy for AML

HHT is primarily used in the clinical treatment of AML in combination. In addition to HHT-based chemotherapy regimens, more studies have focused on the clinical application and mechanism of action of HHT in combination with targeted drug therapy such as FLT3 inhibitors, B cell lymphoma-2 (BCL-2) inhibitors, and pan-histone deacetylase (HDAC) inhibitors. The synergistic mechanisms of HHT are also being learned as research advances.

### 3.1. Synergistic targeting of FLT3 pathway

**Figure 3.**
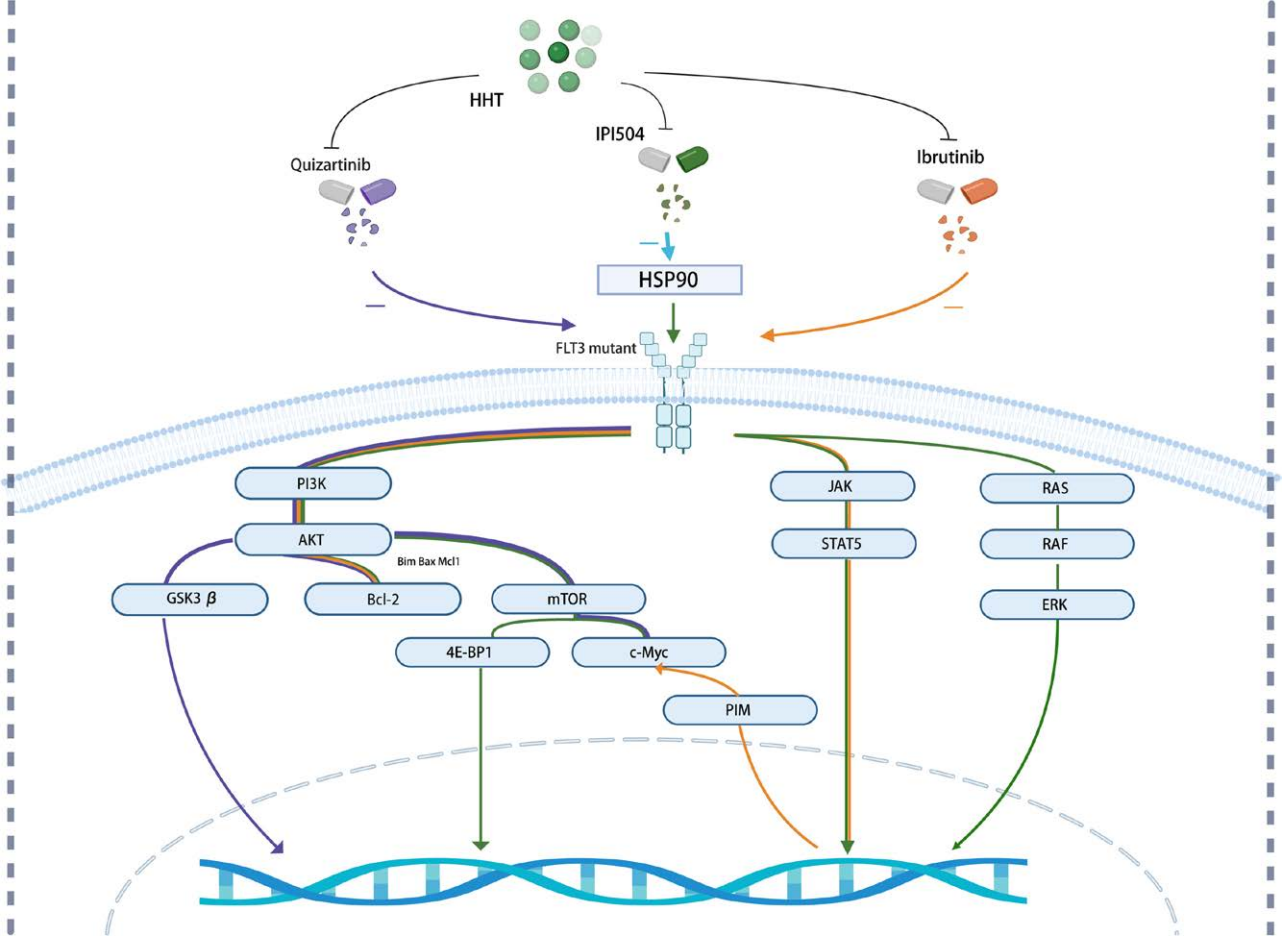
The combination of homoharringtonine (HHT) and quizartinib inhibits the FLT3-AKT signaling pathway and its downstream targets (purple line). The combination of HHT and IPI504 inhibits the FLT3-AKT, JAK-STAT, and Ras-Raf-MAPK signaling pathways and their downstream targets (green line). The combination of HHT and ibrutinib modulates the STAT5/Pim-2/c-Myc signaling pathways, PI3K–AKT signaling pathways, and Bcl-2 family (orange line).

The clinical features and prognosis of AML are significantly influenced by its molecular subtype. About 30% of adult AML patients with a normal karyotype have an FLT3-ITD mutation, which is an internal tandem duplication of the FMS-like tyrosine kinase receptor gene.^[70]^ AML with FLT3-ITD has a poor prognosis, a greater chance of relapsing, and consequently, a worse overall and disease-free survival.^[71]^ To treat AML, it is crucial to specifically block FLT3 kinase activity, and multiple FLT3 inhibitors have been clinically produced. The antileukemia profile of HHT was discovered to correspond with that of FLT3 inhibitors and to preferentially suppress FLT3-ITD AML during the development of an in vitro drug-screening platform. In vitro and in vivo studies have shown that HHT and FLT3 inhibitors function in conjunction.^[72]^ HHT is an essential partner in the therapy of FLT3-ITD AML along with FLT3 inhibition (Fig. 3).

HHT and quizartinib could work together to inhibit FLT3-AKT and its downstream targets GSK3, c-Myc, and cyclin D1, increase the expression of the pro-apoptosis proteins Bax and Bim, and decrease the expression of the anti-apoptosis protein Mcl-1.^[73]^ Most dramatically, sidepopulation and aldehyde dehydrogenase-positive cells, which are allegedly abundant in LSCs, are reduced in quantity when HHT and quizartinib are used in conjunction. Patients with FLT3-ITD AML may benefit from treatment with HHT with quizartinib.

Previous research has demonstrated that FLT3’s molecular chaperone, heat-shock protein 90, is substantially expressed in FLT3-ITD (+) AML and other malignancies.^[74,75]^ In vitro and in vivo studies on FLT3-ITD (+) AML revealed a strong antileukemic effect when HHT and the heat-shock protein 90 inhibitor IPI504 were combined.^[7]^ Mechanistically, apoptosis and cell arrest at G1 were brought on by the synergistic inhibition of FLT3 protein and its downstream AKT, STAT5, ERK, and 4E-BP1 by HHT and IPI504.

It was observed that HHT and ibrutinib were found to have a synergistic inhibitory effect that prevented proliferation, induced apoptosis, and arrested cell cycle at the G0/G1 phase in MV4-11 and MOLM-13 leukemia cells.^[76]^ The results indicate that the primary mechanisms underlying the combination effect involve modulating the AKT pathway, Bcl-2 family, STAT5/Pim-2/C-Myc pathway, activating p21WAF1/CIP1, and suppressing the CCND/CDK complex protein. It is interesting to note that FLT3 and BTK were required for the synergistic cytotoxicity of ibrutinib and HHT.

Collectively, the results showed that combining HHT and FLT3 inhibitor therapy may be a potential treatment option for AML patients, particularly those who have FLT3-ITD.

### 3.2. HHT synergistic BCL-2 inhibitors

BCL-2 is an anti-apoptotic agent that stabilizes the mitochondria and prevents the activation of the proteins that promote apoptosis.^[77]^ Since BCL-2 is dysregulated in AML and its overexpression is what causes treatment resistance and a poor clinical outcome, it is a prospective therapeutic target.^[78,79]^ The expression of Bcl-2 is necessary for AML cells to survive.^[80]^ Bcl-2 protein overexpression, which is associated with chemotherapy resistance, shields cells from apoptosis.^[81,82]^ Therefore, inhibiting anti-apoptotic Bcl-2 family members is hence potential for treating AML.

**Figure 4.**
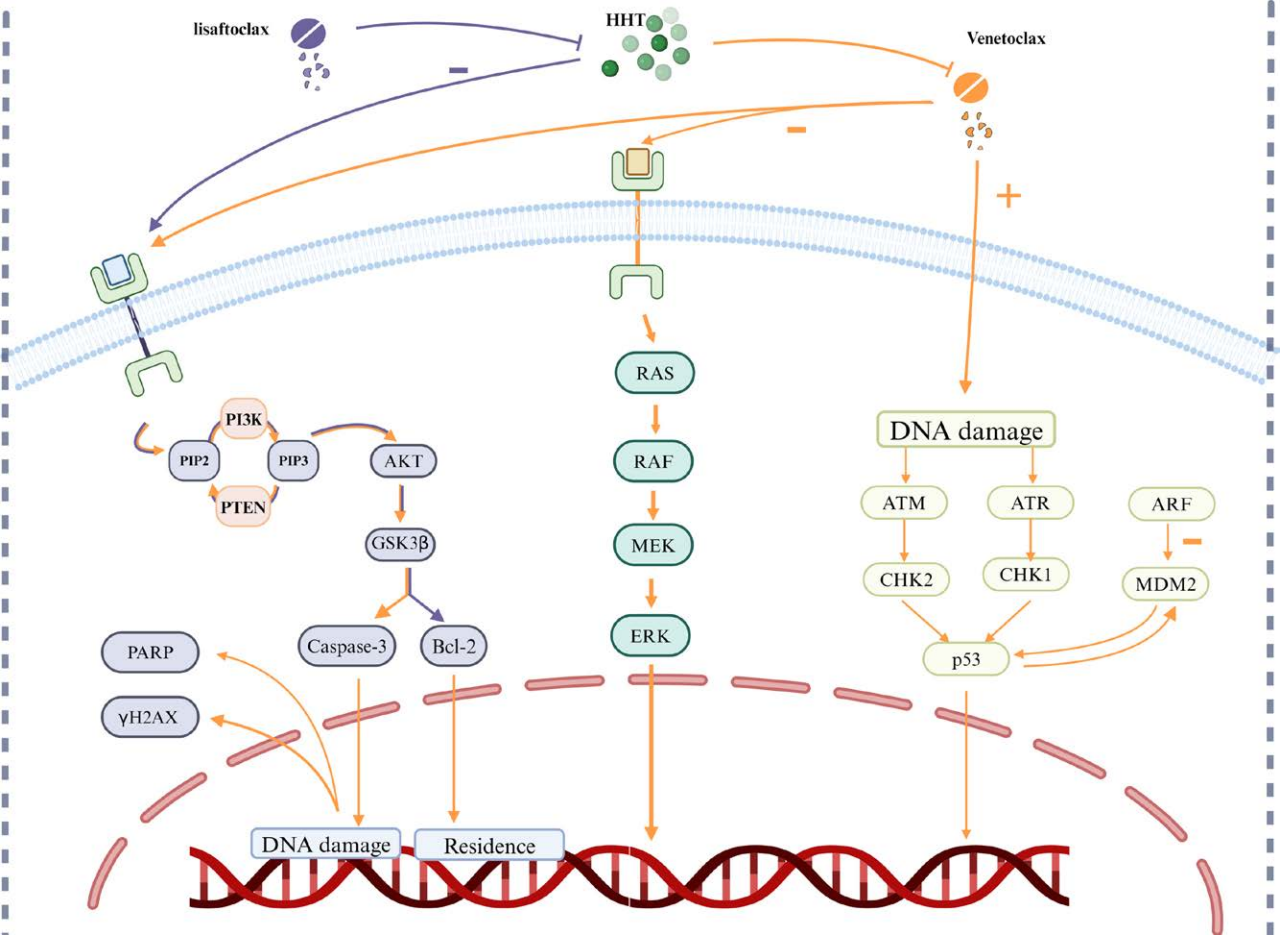
Homoharringtonine (HHT) enhances the proapoptotic effects of venetoclax by inhibiting the MAPK/ERK and PI3K/AKT pathways and activating the p53 pathway (orange line). HHT overcomes the resistance of APG-2575 caused by overexpression of MCL-1 by inhibiting the PI3K/AKT/GSK3 signaling pathway, thereby suppressing it (purple line).

The first high-affinity BCL-2 inhibitor to be discovered was ABT-737. Early research using ABT-737 investigations shown that significant subgroups of AML rely on BCL-2 for survival.^[83]^ A novel BCL-2 homology domains 3 mimicking drug is called ABT-199. ABT-199 was reported to kill LSCs, and in vitro studies have demonstrated its extraordinary antileukemic efficacy in either chemotherapy-sensitive or chemotherapy-resistant AML cells.^[84]^ The findings initiated the study of venetoclax in AML. As a specific Bcl-2 inhibitor, venetoclax effectively induces apoptosis in vitro in cancer cells that overexpress Bcl-2.^[85]^ In a time- and concentration-dependent way, Yuan et al found that venetoclax and HHT worked in concert to suppress AML growth, lower mitochondrial membrane potential, and accelerate AML cell death. When venetoclax and HHT were coupled, the expression of the caspase-3, Poly (ADP-ribose) polymerase, and H2AX proteins elevated. By downregulating the expression of Mcl-1, HHT increased the proapoptotic action of venetoclax. AML cells in the G1 phase of the cell cycle were inhibited by HHT.^[86]^ By blocking the MAPK/ERK and PI3K/AKT pathways and activating the p53 pathway, HHT improved the proapoptotic effects of venetoclax (Fig. 4).

APG-2575 (lisaftoclax) is a novel BCL-2 selective inhibitor. The research showed APG-2575’s antileukemic efficacy was improved by the addition of HHT in primary and AML cell lines both in vitro and in vivo.^[87]^ Mechanistically, the resistance of APG-2575 caused by overexpressed MCL-1 was overcome by HHT by inhibiting the PI3K/AKT/GSK3 signaling pathway, which is how HHT suppressed it. This caused MCL-1 to be dual phosphorylated and degraded (Fig. 4).

### 3.3. Synergistic targeting of TRAIL signaling pathway

The innovative class of anticancer drugs known as HDAC inhibitors deacetylate both histone and nonhistone proteins and regulate the expression of genes that participate in several kinds of cellular processes, such as differentiation, apoptosis, and autophagy.^[88]^ HDAC inhibitors, including suberoylanilide hydroxamic acid (SAHA), have been shown in in vitro studies to have a dual impact on leukemic cells by inducing apoptosis with high doses and encouraging differentiation with low doses.^[89,90]^ HDAC inhibitors not only modulate gene transcription but also have pleiotropic biological effects that may be beneficial for the eradication of AML cells.

According to reports, the cancer treatment drug tumor necrosis factor-related apoptosis-inducing ligand (TRAIL) has a significant apoptotic effect in cancer cells but not in normal cells.^[91]^ By engaging with its corresponding receptors in cells, death receptor 4 and death receptor 5, TRAIL initiates the death receptor-mediated apoptotic signaling (extrinsic) cascade. Caspase-8 and other downstream caspases become active as a result of this.^[92]^

**Figure 5.**
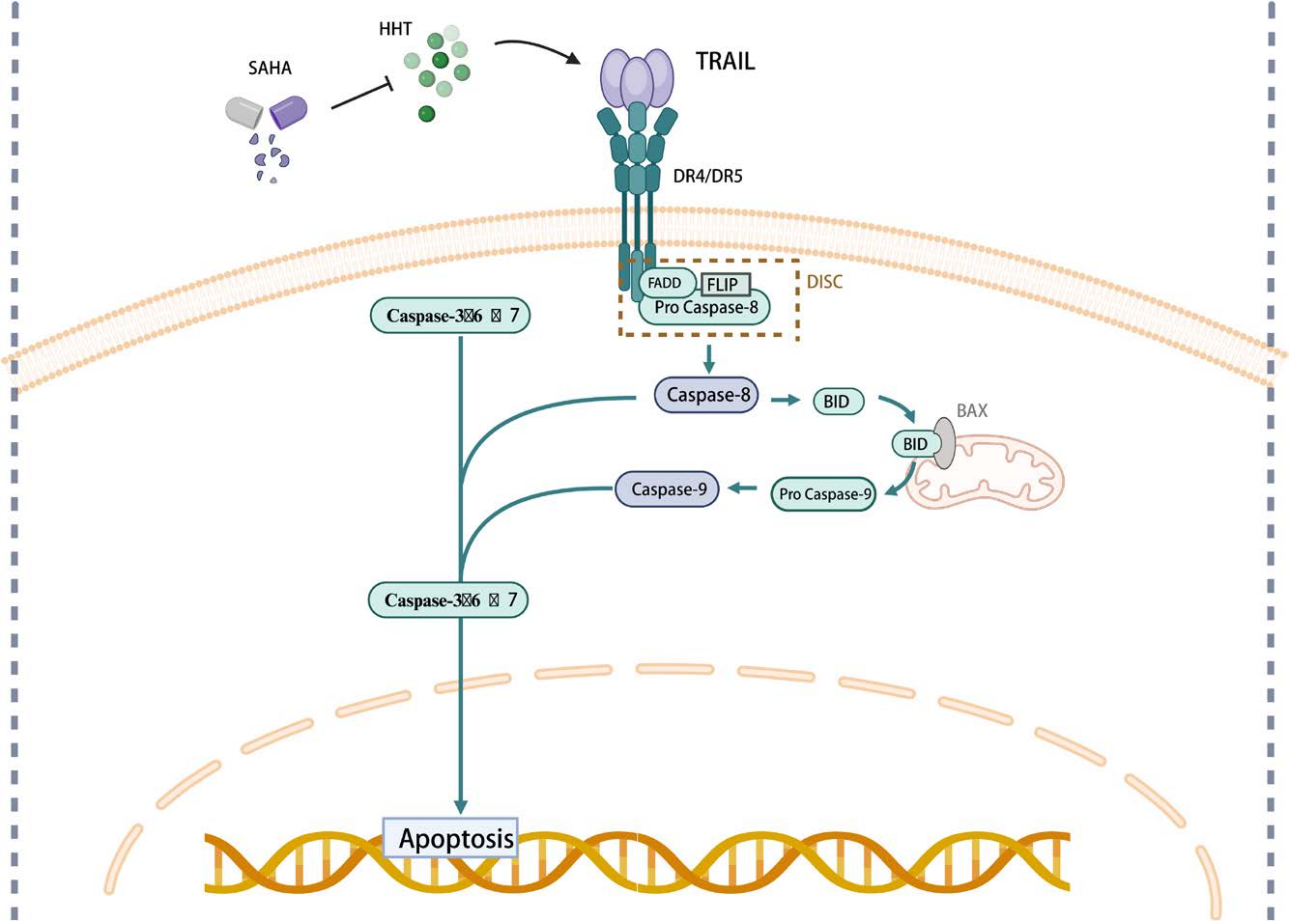
Homoharringtonine (HHT) and suberoylanilide hydroxamic acid (SAHA) together dramatically increased caspase-8 and -9 activation and apoptosis. Notably, HHT dose-dependently increased the expression of tumor necrosis factor-related apoptosis-inducing ligand (TRAIL), although SAHA caused an elevation of death receptor 4 (DR4) and death receptor 5 (DR5).

In a novel approach for treating AML, HHT and SAHA were combined.^[93]^ When HHT and SAHA were combined to induce apoptosis in THP-1 and Kasumi-1 leukemia cells, a synergistic effect was seen. Comparing therapy with each drug alone to the combination, it was discovered that the activation of caspase-8 and -9 was dramatically increased. Notably, SAHA raised the expression of death receptor 4 and death receptor 5, but HHT dose-dependently elevated the expression of TRAIL (Fig. 5). A particular anti-TRAIL antibody was also used to partially prevent the synergistic action of HHT and SAHA. Additionally, leukemia xenograft development in vivo was found to be considerably inhibited by the combined therapy, with increased apoptosis. These findings suggest that a potent therapeutic strategy for the treatment of AML can be achieved by administering HHT at low doses in conjunction with SAHA by controlling the production of TRAIL and activation of the TRAIL apoptotic pathway.

## 4. Discussion

HHT, an alkaloid extracted from cephalotaxus, plays a role in the treatment of AML through varieties of complex mechanisms. HHT has been shown in multiple studies to inhibit cell proliferation and induce apoptosis in AML cells. Additionally, HHT exerts significant synergistic effects when used in conjunction with other chemotherapeutic or targeted medicines. Despite the potential of HHT in the treatment of AML, the issue of drug resistance remains a major challenge, including primary resistance and acquired resistance, the mechanisms of resistance are complex and may involve a variety of molecular pathways and genetic variants that have not yet been fully elucidated.

Genomics biomimicry: using genomics biomimicry in conjunction with patient biomarkers to predict response to HHT and recommend new treatment options for drug-resistant AML patients. Precision medicine: personalized treatment regimens based on patients’ genomic profiles and resistance mechanisms to improve efficacy. Development of new drugs or combination of HHT with other drugs to improve efficacy and overcome resistance, as well as further investigation of the molecular mechanism of action of HHT. Our research team is dedicated to studying leukemia drivers and has identified the UBA2-WTIP fusion gene as a key target. Concurrently, we will examine the effects of HHT on this gene.^[94]^ Subsequent research endeavors should concentrate on demystifying these facets, with the ultimate aim of enhancing the response rate and survival rates of AML patients undergoing HHT-based regimens.

## Author contributions

**Conceptualization:** Haifeng Zhuang.

**Project administration:** Haifeng Zhuang.

**Writing – original draft:** Siyu Shen.

**Writing – review & editing:** Siyu Shen.
